# Supplementary material for: Detailed molecular and epigenetic characterization of the pig IPEC-J2 and chicken SL-29 cell lines
Source: iScience. 2023 Feb 20;26(3):106252. doi: 10.1016/j.isci.2023.106252 (PMC10018572; doi:10.1016/j.isci.2023.106252)
Supplement: Data S1. Complete homer output for identified motifs in Pig IPECJ-2, related to Table 2 — Homer motif analysis results for histone modifications H3K4me1, H3K4me3, H3K27ac, and enhancer elements of pig IPECJ2 cell line. P-values >1e-10 are possible false positives. Within each folder (e.g. peak_files_CTCF) are the html files showing the identified motifs when using homer (e.g. homerResults.html). [file mmc2.zip › S5/Pig_IPECJ_2/peak_files_H3K4me3/homerResults/motif9.similar.html]

motif9

## Information for motif9

T
G
A
C
A
T
G
C
T
A
C
G
T
G
A
C
C
T
A
G
A
C
T
G
G
C
A
T
C
G
A
T
  
Reverse Opposite:  

C
G
T
A
C
G
T
A
G
T
A
C
G
A
T
C
A
C
T
G
A
T
G
C
T
A
C
G
A
C
T
G
  

|  |  |
| --- | --- |
| p-value: | 1e-102 |
| log p-value: | -2.352e+02 |
| Information Content per bp: | 1.630 |
| Number of Target Sequences with motif | 5844.0 |
| Percentage of Target Sequences with motif | 26.47% |
| Number of Background Sequences with motif | 5409.2 |
| Percentage of Background Sequences with motif | 20.44% |
| Average Position of motif in Targets | 151.5 +/- 84.7bp |
| Average Position of motif in Background | 148.7 +/- 106.7bp |
| Strand Bias (log2 ratio + to - strand density) | 0.0 |
| Multiplicity (# of sites on avg that occur together) | 1.11 |
| Motif File: | file (matrix) reverse opposite |

### Similar de novo motifs found

|  |  |  |  |  |  |  |  |
| --- | --- | --- | --- | --- | --- | --- | --- |
| Rank | Match Score | Redundant Motif | P-value | log P-value | % of Targets | % of Background | Motif file |
| 1 | 0.623 | G C A T G T C A A T G C A G T C A T G C C T A G A C T G G A C T G A C T A G T C | 1e-72 | -167.898007 | 32.31% | 26.80% | motif file (matrix) |
| 2 | 0.605 | G C T A A T G C A G C T A C T G A T G C T A C G A C G T A C G T A G T C A G T C | 1e-57 | -132.743743 | 9.55% | 6.69% | motif file (matrix) |
